# How confidence in health care systems affects mobility and compliance during the COVID-19 pandemic

**DOI:** 10.1371/journal.pone.0240644

**Published:** 2020-10-15

**Authors:** Ho Fai Chan, Martin Brumpton, Alison Macintyre, Jefferson Arapoc, David A. Savage, Ahmed Skali, David Stadelmann, Benno Torgler

**Affiliations:** 1 School of Economics and Finance, Queensland University of Technology, Brisbane, Australia; 2 Centre for Behavioural Economics, Society and Technology (BEST), Brisbane, Australia; 3 Newcastle Business School, University of Newcastle, Callaghan, Australia; 4 Deakin University, Burwood, Australia; 5 University of Bayreuth, Bayreuth, Germany; 6 CREMA—Center for Research in Economics, Management, and the Arts, Zürich, Switzerland; Middlesex University, UNITED KINGDOM

## Abstract

Confidence in the health care system implies an expectation that sufficient and appropriate treatments will be provided if needed. The COVID-19 public health crisis is a significant, global, and (mostly) simultaneous test of the behavioral implications arising from this confidence. We explore whether populations reporting low levels of confidence in the health care system exhibit a stronger behavioral reaction to the COVID-19 pandemic. We track the dynamic responses to the COVID-19 pandemic across 38 European countries and 621 regions by employing a large dataset on human mobility generated between February 15 and June 5, 2020 and a broad range of contextual factors (e.g., deaths or policy implementations). Using a time-dynamic framework we find that societies with low levels of health care confidence initially exhibit a faster response with respect to staying home. However, this reaction plateaus sooner, and after the plateau it declines with greater magnitude than does the response from societies with high health care confidence. On the other hand, regions with higher confidence in the health care system are more likely to reduce mobility once the government mandates that its citizens are not to leave home except for essential trips, compared to those with lower health care system confidence. Regions with high trust in the government but low confidence in the health care system dramatically reduce their mobility, suggesting a correlation for trust in the state with respect to behavioral responses during a crisis.

## 1. Introduction

During the first several months of 2020, most countries attempted to implement various social distancing measures and other non-pharmaceutical interventions (NPIs) to combat the spread and transmission of COVID-19; for example, bans on large gatherings, closure of schools, gyms, bars and restaurants, and mandating stay-at-home policies (otherwise known as shelter-in-place orders) with varying degrees of strictness. The literature is still in its infancy regarding the direct effect of NPIs on the spread of COVID-19 –and more importantly–on their efficacy in containing infectious disease spread [1–4]. The overall health, societal, and economic effects of these measures are not yet well-understood.

The observed levels of cooperation and compliance with government measures have been attributed to a combination of extrinsic motivations such as social pressure [5], intrinsic motivations that include moral support and social norms [6–15], and are dependent on factors such as local income, risk-taking behavior, personality characteristics, political orientation, trust in the government, trust in media sources, and belief in science [16–23]. Relatively early in the pandemic, Van Bavel et al. [24] pointed out the path forward for several of these behavioral interventions. Their overview of knowledge in social and behavioral science explored the potential avenues and channels through which these disciplines can support real-time public health crisis management. Many of the authors’ suggestions are reflected in current policy, and many points remain as further opportunities to improve policy responses.

During a pandemic such as COVID-19, confidence in the health care system is a prominent concern for citizens. Thus, we analyze whether societies with lower confidence in the health care system respond with precautionary reduction of mobility to protect themselves from the uncertainty inherent in responses to and treatment of the novel coronavirus. Confidence that “you will be adequately treated when you are in need of health care” is related to trust in the health care system [25, p227]. Specifically, confidence is an expectation of the competence and capacity of the system based on experience and reason; indeterminacy is calculable due to the determination of risk [26]. On the other hand, as discussed further in Section 2.4, trust involves a moral dimension wherein indeterminacy is incalculable due to uncertainty [26]. We hypothesize that regions with low confidence in the health care system will exhibit a stronger behavioral reaction to the outbreak (evidenced by a decrease in social mobility due to staying at home) than will regions with higher confidence. More confidence in the health care system may also encourage individuals to seek treatment (earlier) and therefore potentially improve societal outcomes [27], whereas a lack of confidence may result in avoidance until all other options are exhausted.

## 2. Data

### 2.1 Mobility

We use mobility measures at the country and regional level from the COVID-19 Community Mobility Reports [28], accessed on June 10, 2020 [29]. The data are anonymized and aggregated, generated from the activity of Google users who have opted into the location history service. Google provided records of percent change in total number of visitors to locations classified as Retail & Recreation, Grocery & Pharmacy, Parks, Transit Stations, Workplaces, and percent change in length of stay at Residential places within the geographic area, from 15 February to 5 June 2020. For privacy reasons, Google omits values where the traffic volume is not high enough to ensure anonymity.

Mobility change is calculated as the percentage difference from the median value of the same day of the week between 3 January and 6 February 2020 for each corresponding location. While the Community Mobility Reports are available for 135 countries and for more than 1,800 sub-national regions (excludes US counties), our sample is restricted to the number of countries for which we have data on the level of confidence in the health care system. The measures are only available for 38 European (or Eurasian) countries; therefore, these territories form the initial sample of this study. Mobility is recorded on the sub-national level (621 regions) for 28 out of the 38 countries, while the rest are measured on the country level (S1 Table). To increase statistical power, we combine observations from the two levels in the analysis.

For this study, we focus on the change in the duration of time at home as the main variable capturing the behavioral reaction to the pandemic. As a robustness check, we also conduct an analysis using the average percentage change in travel to the five non-residential locations (presented in the Supporting Information). The results of non-residential mobility are–as expected–a mirror-image of the behavioral changes observed in the main analysis.

### 2.2 Confidence in the health care system

As discussed previously, measures of confidence and trust in the health care system and other public service institutions are sometimes closely connected. We distinguish between confidence as an experience-based calculation of competency in a health care system, and the moral dimension of trust in the institution to ‘do the right thing’. For example, Bunton and Gilding [30] studied the mass adoption of Human Papilloma Virus (HPV) vaccination in Australia, finding that confidence in the vaccination regime is transferred from the accepted ‘truism’ of a childhood vaccination program and resultant confidence in the process. However, confidence in that system is not the same as trust, as participants were cynical and distrustful of the vaccine marketing and lack of informed consent within focus groups; demonstrating the dynamics of confidence in the expertise of the system, but not trusting in the motives of the regime or institution.

Existing institutional conditions are tested by new challenges, and the COVID-19 crisis has tested the capacity of global health systems. Evidence from health care responses in China indicate that health care providers were nervous and lacked self-confidence in their ability to cure patients affected by this new disease; the risks, transmissibility, pathogenicity, and possible treatments were not well understood [31]. In addition, a lack of experience among health care workers in intensive care (e.g., dealing with technological procedures such as mechanical ventilation) generated additional uncertainty. Team members from different specialties were required to work together, increasing transaction costs in groups that were accustomed to different protocols and skills [31]. Such issues have appeared all around the world.

A patient may not know the specific quality of care they will receive from an individual doctor [25] under normal circumstances, but does know in general the average quality of care they would receive in the health system; for example, a patient with a broken leg or high blood pressure has some ‘common’ knowledge or expectations about the outcome. However, given the extreme uncertainty inherent in a pandemic involving a new virus for which every treatment is experimental, a patient cannot know if any treatment has a chance of being effective. This complicates the dimensions of uncertainty beyond the credence good problem [32], and beyond the usual challenges involved in maintaining confidence in the health care system. A sense of confidence in the health care system, in general, will also affect whether people are compliant with the recommendations of the health system [33], and–as mentioned previously–whether and when they seek treatment if they are ill [25].

Our measure for health care system confidence comes from the European Values Survey (EVS) and is derived from the question: “I am going to name a number of organizations. For each one, could you tell me how much confidence you have in them: is it a great deal of confidence, quite a lot of confidence, not very much confidence or none at all? (Health Care)” (see https://europeanvaluesstudy.eu/methodology-data-documentation/). The four values in the Likert scale are recoded in reverse for intuitive and quantitative purposes, i.e., “none at all” took a value of 4, with “a great deal” taking a value of 1. For each country, we take the latest survey wave that included this question. Values that were unknown or were left unanswered are coded as missing and excluded from the analysis. For the 38 countries, 27 (71.05%) are taken from the 2017 survey wave, 10 (26.32%) from the 2008 survey wave, and 1 (2.63%; Turkey) from the 1999 survey wave (S1 Table). We then calculate both country and regional (Nomenclature of Territorial Units for Statistics division 2 (NUTS2) averages.

At the country level, the Spearman rank correlation between confidence in the health care system in the latest (5^th^; 2017) wave and in the 4^th^ wave (2008) is very high with *r* = 0.78, *p* < 0.001, n = 32 countries. The correlation between the 5^th^ and 3^rd^ wave is also quite high; *r* = 0.72, *p* < 0.001, n = 23 countries. Similarly, at the regional level, we also find higher rank correlations between the 5^th^ wave and two earlier waves with *r* = 0.8, *p* < 0.001, n = 162 regions (4^th^ wave) and *r* = 0.54, *p* < 0.001, n = 79 regions (3^rd^ wave), respectively. Additionally, we did not find that country averages systematically change over time (paired t-test indicates the mean in confidence did not change from the 3^rd^ to 4^th^ or 5^th^ wave, nor does it change from the 4^th^ to 5^th^ wave, p > 0.1 with a two tailed test); however, we did find that the regional average decreases from 4^th^ to 5^th^ wave (by ~3% or 0.07 on a 4 point scale, p < 0.01). We therefore provide an analysis of the main results in the Supporting Information to include only those countries (*n* = 27) for which there is an available 2017 EVS survey providing confidence in the healthcare system. The results do not change qualitatively to any extent (S7–S10 Figs).

### 2.3 Controls

We incorporate several relevant control variables; first, as mobility patterns differ across weekdays and weekends, a binary variable is included to signify whether the day is a weekend according to each country’s definition (ddefinition of weekend is based on https://en.wikipedia.org/wiki/Workweek_and_weekend (accessed 07 May 2020)). An indicator variable denoting the periods before and after the WHO declared a world-wide pandemic on 12 March 2020. Second, a set of government response indicators (recorded daily at the country level) of closures and containment relating to schools, workplaces, public events, private gatherings, public transport, residential confinement, and domestic travel are captured from the Oxford Covid-19 Government Response Tracker (OxCGRT) as they have a direct impact on reducing mobility. Each indicator categorizes the level of strictness of the respective policy on an ordinal scale (descriptions of level of strictness are provided in detail in the Codebook for the Oxford Covid-19 Government Response Tracker (version 2.1, accessed 12 May 2020)). From the OxCGRT database, we also obtain the daily record of the number of Covid-19 related deaths and confirmed cases, taken from the European Centre for Disease Prevention and Control (ECDC) and from the Johns Hopkins University Center for Systems Science and Engineering (JHU CSSE) data repository. We derive the number of days since or before the first confirmed death in the country and number of confirmed cases (in natural log plus 1) as controls.

We also include the country’s socioeconomic and demographic structure to control for the elasticity in mobility change, for example, mobility in less developed countries might be less likely to change as workers (with labour intensive jobs) are not easily able to work from home [34]. Similarly, countries with a larger proportion of their population residing in urban areas or those with a higher population density might have more elastic mobility as daily supplies or social network support are more easily accessible [35]. We therefore include log GDP per capita (constant 2010 USD), unemployment rate (% of total labor force), population density (people per squared km of land area), percentage of urban population, share of population over 65, and percentage of females in the population as obtained from the latest record from the World Development Indicators (WDI), as well as the average household size obtained from the United Nations Household Size & Composition 2019 report. In addition, we include the Education index (ranging from 0 to 1; calculated using mean and expected years of schooling) from the Human Development Reports (United Nations) as a measure of human capital, and control for corruption risk rating (ranging from 0 to 6, with 6 being least risk in corruption) from the International Country Risk Guide (ICRG). Furthermore, to control for the country’s health care capacity and structure, we use the number of hospital beds per thousand and the percentage of out-of-pocket expenditure as total household expenditure on health) as a proxy.

Lastly, as the time period of our sample spans over four months, changing seasons and weather conditions are likely to affect daily mobility [28, 36]. We thus control for daily maximum and minimum temperature (to the tenths of Celsius (°C)) on the regional level, obtained from the Global Historical Climate Network Daily (GHCN) database. For each region, we take the average temperature records from all weather stations located within 50km from the centroid of the region, with x-y coordinates of each area’s centroid obtained from GADM.org.

Accounting for missing control variables, our most restrictive sample (including all control variables) includes 591 geographical units from a total of 32 countries, with 24 countries at the subnational region levels (579 regions) and seven countries at the national level.

### 2.4 Generalized trust, trust in the state, and governance quality

If confidence in the healthcare system is not necessarily the same as trust in the healthcare system [26, 30], this is especially true with respect to trust in government or other institutions. When studying trust and institutions, the literature usually distinguishes between horizontal trust and vertical trust; the former being trust between citizens, whereas the latter is trust between citizens and authorities [37, 38]. Higher levels of generalized trust in formal institutions can facilitate policy compliance and reduce transaction costs in coordinating responses to problems at the state level. At the same time, higher levels of trust between citizens can contribute to social capital, facilitating more effective collective action outcomes at the grassroots level [39]. However, these two dimensions of trust are not independent, and especially where formal institutions fail, there is often a role for informal institutions, social capital, and voluntary coordination to address problems facing the community [39].

In our study, confidence in the institution is measured at the organizational level. This question does not ask about specific different dimensions of confidence, but rather about the institution or organization of health care generally. To complicate matters further, trust in the health care system itself can be distinctly different from trusting individual medical practitioners. For example, Calnan and Sanford [40] surveyed England and Wales for both types of trust, finding that at the time, trust in doctors was quite high, while there were very low levels of trust in the organization and funding of the health care system at the institutional level. Generalized trust helps manage uncertainty about the quality of care received in the health care system from individual practitioners, and from a sociological perspective, trust in the system substitutes for being able to trust specifically in care from a doctor [41].

If generalized institutional trust represents trust in the system at large, perceived corruption represents the opposite. The pervasive lack of trust engendered by corruption damages compliance in general, as it “involves the capture of political and economic power for the elites and for those on the ‘inside’ of the circle of influence, destroying morale, reciprocity and the motivation to take collective action in providing any public good” [39, p45]. This can lead to inefficiencies, reorganization of institutional structures, decreased governance, breakdown in law and order, development of black markets, and overall reduced compliance with policies enacted by the state.

Over the past several decades, researchers have noted a decrease of generalized trust in institutions [42]. Such changes have not been uniform across all institutions, as some have seen improved levels of trust while others have experienced decreased levels of trust [42]. Thus, confidence in the health care system at the organizational level is different from trust in the state, or trust in all institutions within that country. A recent study by Zhao et al. [43] finds that trust in the health care system is positively related to the GNI per capita and health care expenditures of the country (with the USA serving as a notable exception, exhibiting high levels of expenditure and low levels of trust). Institutional conditions are different for different countries; a fact our analysis explicitly controls for with country fixed effects. However, it is important to disentangle the level of confidence in the health care system from the trust in the state in general–its institutional framework and hierarchy–by controlling for perceived corruption or other governance factors, as this could undermine public institutions, or introduce mediating effects on compliance.

In addition to perceived corruption, our proxy of broader institutional conditions employs components of the ICRG political risk rating as measures of governance quality; namely *Bureaucracy Quality*, *Democratic Accountability*, *Government Stability*, and *Law & Order*. For generalized trust, we use the trust measure from the Global Preference Survey [44, 45] aggregated at the region average. We also derive two additional measures of trust based on the items *Most people can be trusted* and *Trust*: *Other people in country* from the last available wave of the World Value Survey. Lastly, to measure trust in government institutions, we utilize the question on the level of confidence in *Government*, *Parliament*, *Political Parties*, *Civil Services*, and *Justice System/Courts* from the WVS. Again, we aggregate the measures into region average, and for ease of comparison, we standardize each of these variables at the regional level.

## 3. Methods

**Fig 1:**
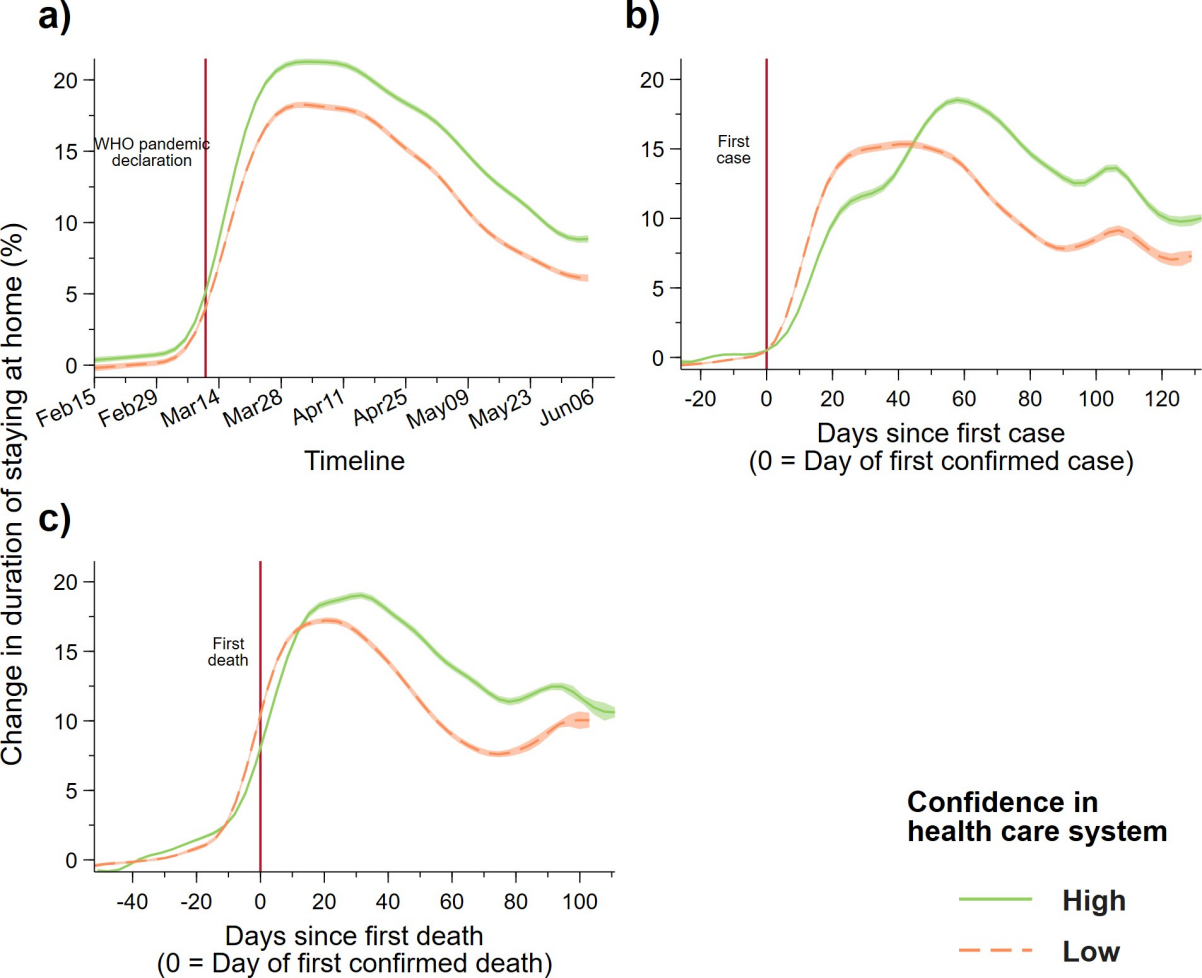
Increase in duration of staying home (daily) by regions with high (*n* = 386) and low (*n* = 379) levels of confidence in the health care system (a) over time (from 15 Feb to 5 June), (b) since the first confirmed case in the country, and (c) since the first confirmed death. The high and low confidence groups are defined as regions with average levels of confidence in the health care system that are higher or lower than the median value of all regions. We applied local mean polynomial smoothing (Gaussian kernel function with a bandwidth of 5 days) to the daily mobility changes of the two groups with 95% confidence interval. Observations from regions are given weights equal to the inverse of the number of regions of the country, observations from countries are given weight equal to 1. Vertical line in panel a) represents the date on which the World Health Organization (WHO) declared COVID-19 as a pandemic.

To look at the way in which citizens’ trust in the health care system translates into different behavioral reactions to the pandemic outbreak, we first show the change in mobility patterns separated by regions whose average level of confidence in the health care system is above (HIGH) or below (LOW) the median value (Fig 1). To ensure equal representation for each country due to the varied number of regions, we apply weights to each regional observation that is inverse to the number of regions of the corresponding country.

Next, we estimate the effect of confidence in the health care system on changes in mobility due to the outbreak using a regression approach. Specifically, we estimate the following model:

$$\begin{array}{l} Mob_{r,\;t}=\alpha +\varphi Conf_{r}+\delta Covid_{r,t}+\gamma \left(Conf_{r}*COVID_{r,t}\right)+\\ controls+D_{week}+D_{cty}+\varepsilon_{r,t} \end{array}$$

where *Mob* is mobility measure (duration of staying home compared to baseline period) of region *r* at time *t*, *Conf* is the measure of confidence in the health care system in region *r*, and *Covid* the number of weeks since the first confirmed Covid-19 case or related death occurred in the country of region *r*. *D*_*week*_ and *D*_*cty*_ represent dummy variables for calendar week and country, correspondingly. We include the controls, time dummies and country dummies procedurally to demonstrate the sensitivity of the estimation results. The time and country fixed-effects respectively control for time-varying confounding factors affecting all regions and country-specific confounders. Note that time-invariant country-specific control variables are removed from the model when country-fixed effects are included. As *Conf* was measured at the region level, we cluster the standard errors accordingly. All statistical analyses were performed using Stata/MP 16.1.

## 4. Results

In Fig 1A, we demonstrate that the change in the duration of staying home (relative to a baseline period in January) remained relatively flat during early February and gradually increased from the beginning of March, followed by a rapid surge until the start of April for both high and low confidence groups (the vertical red-dashed line indicates the date that WHO declared COVID-19 a pandemic). Throughout the whole sample period, the magnitude of mobility change seems to be larger for regions with higher levels of confidence in the health care system.

However, when we change the time reference for each region to the number of days since the outbreak in their country, defined as the date of the first confirmed case (Fig 1B) which we normalize to 0, we find that regions with low confidence in the health care system reduce their mobility (increased duration of staying home) much more rapidly than regions with high levels of confidence. This effect is represented by the slope differential between the two curves during the first three weeks (about 21 days) of the outbreak. After this point, the increase in mobility reduction for the low confidence group plateaued and eventually started to decline at around the 60-day mark. During this plateau state, mobility reduction for the high confidence group continued to increase, surpassing the low confidence group before declining at around 60 days since the first confirmed case.

Switching the time reference to the number of days since the first confirmed death in the country revealed a similar (but less stark) pattern between the two groups. When we restrict the sample to only countries with confidence measure from the latest (5th) wave, we find the difference in mobility pattern more visible in relation to after the first cases and death occurred (S7 Fig). Keeping in mind that the mobility pattern started to differ since the first confirmed case, there seems to be a difference in mobility reduction at around the time of the first death in the country.

We found a similar pattern when looking at changes to non-residential mobility; regions with low levels of confidence in the health care system were the first to reduce mobility outside the home after the first confirmed Covid-19 case (S1 Fig). In addition, when we define the high and low confidence group by the region’s value above or below the country average, we find a small significant difference in mobility reduction between the two groups after the first confirmed case (S2 Fig). We check the results by not applying weights to the regional observations (S3 Fig) or using country-level mobility and separating countries into below and above the country median (S4 Fig); in both cases, we find similar outcomes.

**Fig 2:**
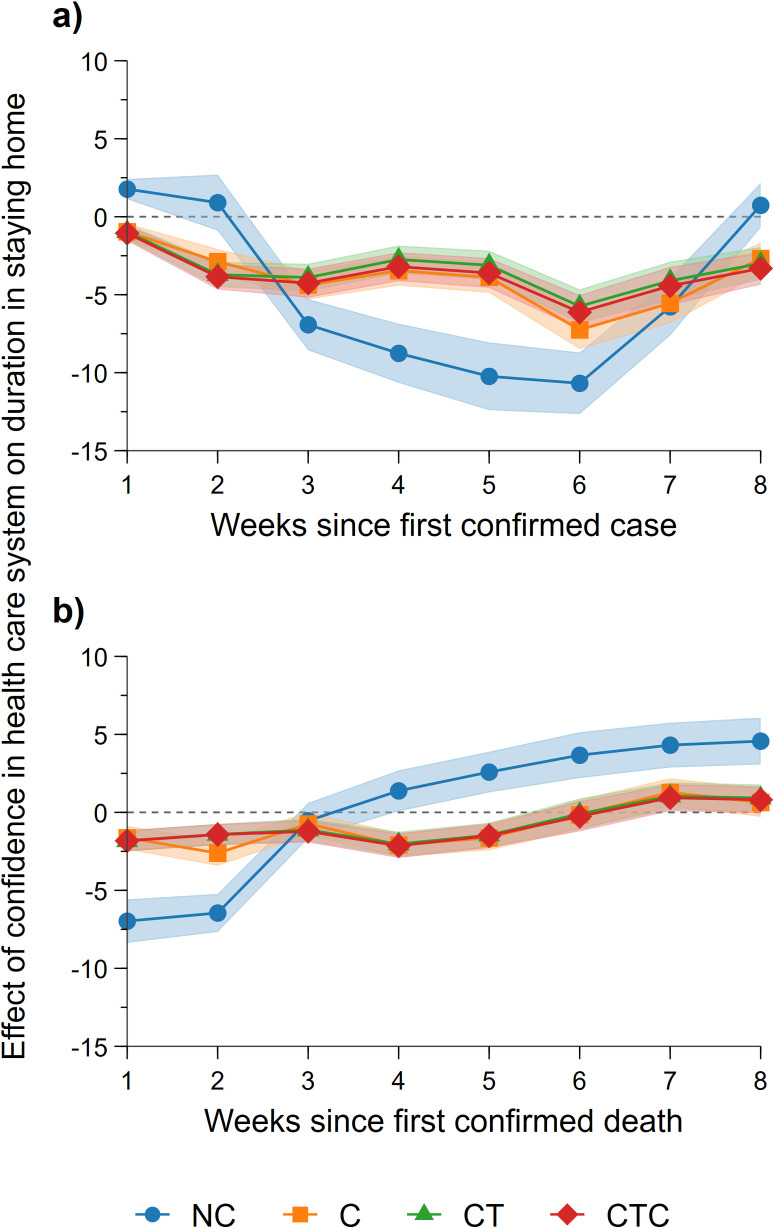
Estimated effect of confidence in the health care system on mobility changes (duration in staying home) since the first confirmed case (a) and death (b) in the country. Estimates obtained from random-effects GLS estimates with no control variables (NC), with control variables (C), with control variables and time-fixed effects (CT), and with controls, time and country-fixed effects (CTC). Areas represent 95% confidence intervals of the effect estimate.

Next, we graphically present a set of regression results to show the effect of confidence in the health care system on mobility variations due to the outbreak, and the dynamics of how such variations change over time (see Fig 2). To do this, we use a random-effects model to regress mobility change on the interaction between the levels of confidence in the health care system and the number of weeks since the first confirmed case or death (the baseline is the time period before the first case or the first death occurred). We report the unconditional results, without control variables, and compare them with a specification controlling for mobility changes due to heterogeneity in social, economic, and population structure, containment policies across countries, and temperature across geographic areas. We also compare the results with the model incorporating time and country fixed effects and report the regression results for the control variables in S2 Table.

The results in Fig 2 support our earlier findings. In the specification with control variables (Model C in Fig 2), we find a strong and significant effect of confidence in the health care system on mobility reduction since the outbreak in the country. Specifically, we observe that since the *second* week of the outbreak (from day 8 to day 14 since the first confirmed case within the country) (Fig 2A), regions with *higher* confidence in the health care system have a *smaller increase* in the duration of staying home after reporting the first confirmed case. The effect is salient when compared to the pre-outbreak period, suggesting a small positive effect of confidence in the health care system on increased mobility. We also note that the confidence effect is not substantially different from 0 in the *first* week since the outbreak occurred, indicating there is a lag in behavioral reaction. In addition, the confidence effect seems to wear off after about eight weeks post-outbreak. While omitting all controls (Model NC) tends to overestimate the confidence effect (both positive and negative direction), the addition of time fixed-effects (Model CT) and time and country fixed-effects (Model CTC) only slightly reduces the estimated effect size, while the statistical significance of the results remains unchanged.

With respect to behavioral reactions since the first reported death in a nation, we find a further difference in mobility reduction between regions with different levels of confidence in the health care system. In particular, the effect of health care system confidence on mobility reduction is greatest during the first seven days after witnessing the first Covid-19 related death (Fig 2B). A similar pattern is revealed in the analysis on countries for which confidence measures were collected in the 5^th^ EVS wave (S8 Fig).

Furthermore, we check the sensitivity of the confidence effect by replacing the corruption index with other institutional and governance quality measures as well as generalized and institutional trust (S5 Fig and S3 Table). The results of our primary finding regarding the effect of health care system confidence on mobility since the first confirmed case remain virtually unchanged. In addition, as reported in S3 Table, we find that the effects of other institutional quality measures on mobility are similar to the results observed on the corruption index, where regions with higher institutional quality exhibit greater reduction in mobility (higher compliance). Yet, regions with lower trust in government, political parties, and the justice system tend to exhibit less reduction in mobility. Lastly, even though the three measures of generalized trust are somewhat positively correlated, their effects on mobility are not conclusive.

**Fig 3:**
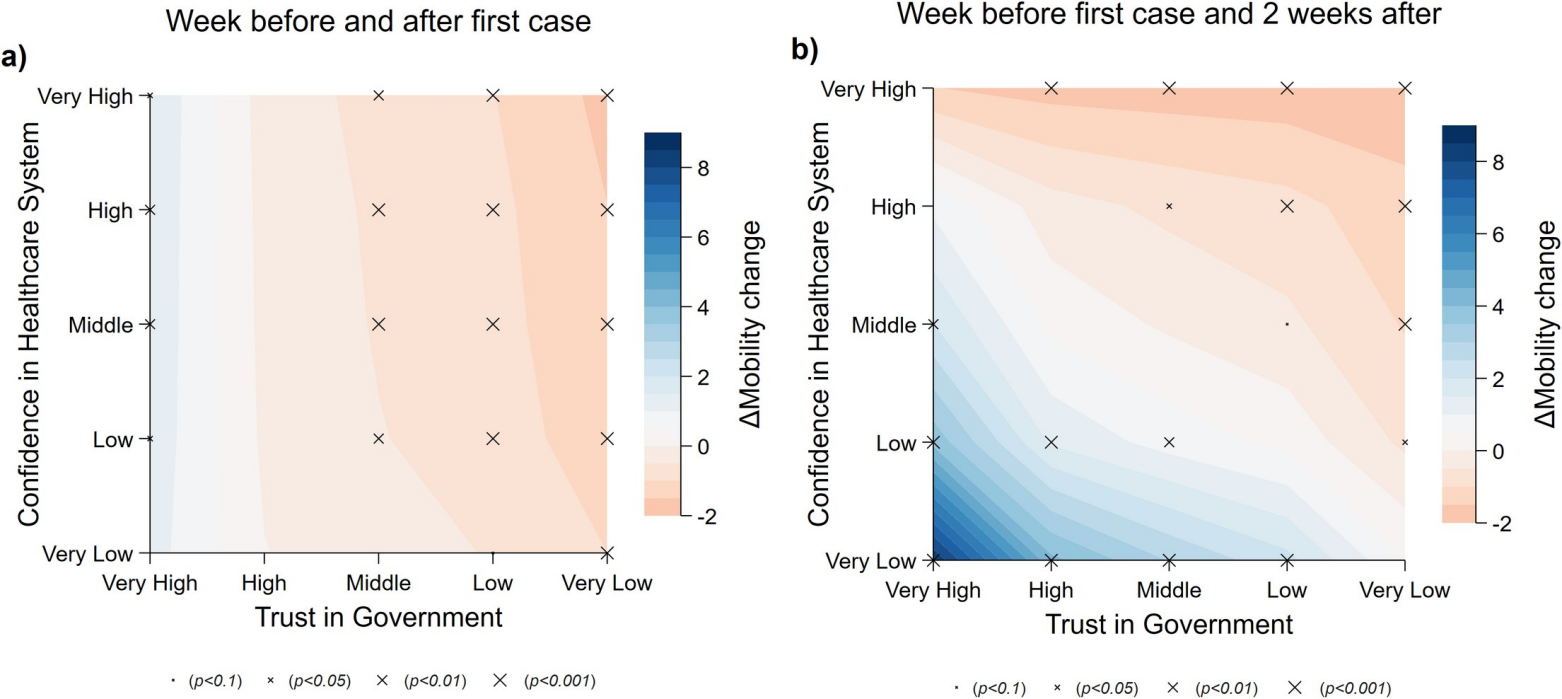
Effect of trust in government and confidence in the health care system on mobility. Blue indicates the estimated reduction in mobility change (difference in percentage increase in the duration of staying home) in the *first* (a) and *second* (b) week since the first confirmed case, compared to mobility *one week before* the first case. Effects are predicted from the model at the 1^st^, 25^th^, 50^th^, 75^th^, and 99^th^ percentiles of the distribution of the two variables trust in government and confidence in the health care system, which we categorized into five levels: *very low*, *low*, *neutral*, *high*, and v*ery high*, respectively. Statistical significance of the effect is indicated by the size of the markers.

Next, we consider whether trust in the government mediates the effects of confidence in the health care system. We hypothesize that regions with low health care system confidence but with a high level of trust in the government are more likely to stay at home following the outbreak. To test for this effect, we regress the mobility change on the triple interaction terms between confidence in the health care system, trust in government, and our time variable. Seeing that the effect of confidence in the health care system is largest in the second week since the outbreak, we use mobility change in the week prior to the outbreak as the reference group and visualize the mediation effect by contrasting the difference compared to the first (Fig 3A) and second (Fig 3B) week of the outbreak. The regression estimates are provided in S4 Table.

Similar to the results above, we did not find a strong effect of confidence in the health care system during the first week of the outbreak, but–compared to the week before the outbreak–we observe a slight reduction in staying home for regions with low trust in government (Fig 3A). In the second week, while observing that regions with low levels of trust in the government tend to increase mobility (spend relatively less time at home) on average, regions with high trust in the government but low confidence in the health care system exhibit a dramatic increase in staying at home. This may be attributable to the citizens’ understanding that their health care system does not have the capacity to offer sufficient care, but they trust that the government has their best interests at heart, and have complied with requests to decrease mobility. For example, comparing the mobility change between a typical region with low confidence in the health care system (25^th^ percentile) and high trust in government (75^th^ percentile) to a similar counterpart with high confidence in the health care system and low trust in government, the former results in a 2.27 percentage point increase in the duration of staying home, compared to the latter.

Furthermore, we consider whether the perceived level of corruption mediates the effect of confidence in the health care system on compliance (S6 Fig). We focus on mobility differences in the second week compared with the week before the outbreak, as this is when the effect of confidence in the health care system is most salient. Similar to the results above, we find that regions with low confidence and high institutional quality (high score on corruption index) (lower right corner) have a small increase in mobility reduction (more compliant). However, we also see that this pattern is reversed in regions with less corruption but high confidence in the health care system (upper right corner), where we observe less compliant behavior (less reduction in mobility). Interestingly, regions with high confidence in the health care system but high corruption also reduce their mobility, compared to the regions with low confidence in the health care system, where mobility was not changed significantly.

**Fig 4:**
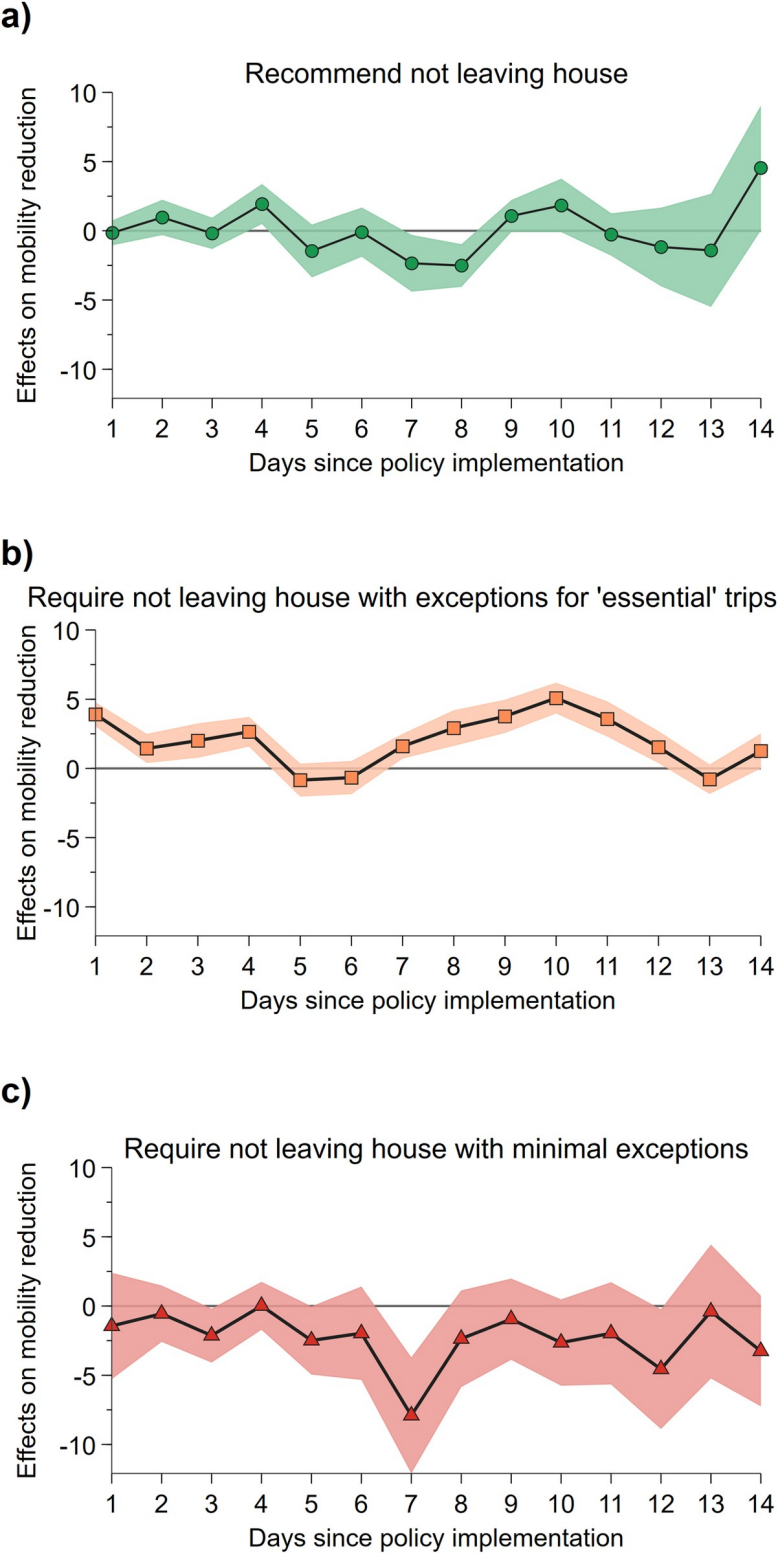
Effect of confidence in the health care system on mobility reduction after introduction of the shelter-in-place policy. We show the effect of health care system confidence on the pre- and post-policy differences to the change in the duration of staying home, over 14 days since the (a) recommendation to stay home, (b) requirement to stay home with exceptions for essential trips, and (c) strict stay-at-home requirement. Areas represent 95% confidence intervals of the effect estimate.

Next, we explore how behavioral reactions differ between regions with high and low confidence in the health care system in response to the introduction of a shelter-in-place policy. To do this, we employ the home confinement policy indicators provided in OxCGRT to pinpoint the date on which the shelter-in-place policy was introduced. In particular, we compare mobility change before and after each of the three policy stages, namely, “recommend not leaving house”, “require not leaving house with exceptions for essential trips” (e.g., exercise, shopping for grocery), and “require not leaving house with minimal exceptions” (e.g., only one person in the household can leave at a time or allowed to leave once a week), respectively. We then assess whether such a difference in mobility change, if any, is related to the difference in the levels of confidence in the health care system by employing a difference-in-difference approach. Specifically, we construct a variable with a value equal to the number of days since the corresponding policy stage was employed and a value of 0 for the time period before that policy stage. We then regress the interaction term of this variable with our measure of confidence in health care on mobility change, 14 days before and after the policy stage was first introduced. We only focus on policies implemented to tighten social confinement. That is, only policy stages that increase the restrictions on movement were included (e.g., going from recommendation stage to a mandate against leaving home, but not the opposite). In addition, we do not include post-policy observations that overlapped with the introduction of a more restrictive policy; e.g., if a country imposed a requirement against leaving home three days after a recommendation, then our analysis on recommendation only includes three post-policy observations for that country. This effectively allows us to assess the post-policy mobility daily changes (in comparison to changes evident just before the policy implementation) due to the effect of confidence in the health care system. We estimated the effect separately for the three policy stages and report the results in Fig 4. In each regression, we included the set of control variables in Model C, trust in the government, and day of the week dummies.

We observe that regions with higher confidence in the health care system are more likely to further reduce mobility once the government mandates that its citizens are not to leave home except for essential trips, compared to those with lower health care system confidence (Fig 4B). We test whether the average of all interaction terms is significantly different from 0 (χ^2^ = 23.2; p<0.001), and employ another specification where we use a post-policy dummy instead of daily dummies (z = 5.88; p<0.001). In both cases, the result suggests the effect is highly significant.

We find that, on average, the post-policy stay-home duration difference is 1.96 percentage points higher for a 1 unit increase in health care system confidence over the 14 days. In particular, we can see that the effect is already highly apparent in the first few days of the policy introduction. However, we did not observe any post-policy differences in mobility change between regions with dissimilar levels of confidence in health care; this is the case for both recommendations (Fig 4A) and the more restrictive stay-at-home requirement (Fig 4C). For both policy stages, neither the average of the interaction terms nor the post-policy dummies are significantly different from 0. Nevertheless, when repeating the analysis using a sample that excludes countries for which confidence measures were not captured in the 5^th^ wave of EVS, we find that mobility further reduces after the highest level of restriction is in place (S10C Fig).

## 5. Conclusion

The current study has demonstrated–through the use of a mobility proxy–that public confidence in the health care system is positively related to differences in compliance behavior during a pandemic crisis situation. Moreover, we show that mobility changes with respect to confidence levels also vary across time periods, i.e. days since the first known case and days since the first known death. The most interesting aspect of our time-dynamic analysis is the finding that societies with low levels of health care confidence initially exhibit a more significant response and stay home, but over time this reaction plateaus more quickly than does mobility in high health care confidence societies, and also declines with greater magnitude. Less confidence in the health care system may signal citizens’ awareness that the health care system cannot handle an outbreak–or even that their own access to health care is not guaranteed–leading to rapid and extensive self-isolation. The question of why we observe a faster drop in compliance for low-confidence societies is more difficult to understand; however, there are several potential reasons to consider. Countries with lower health care quality may need more time to handle a health care shock such as COVID-19 in terms of securing a supply of equipment or qualified staff members. Citizens may initially restrict their movement, due to awareness of insufficient personal protective equipment (PPE) or specialists, and then start to increase their mobility once the health care system is better able to cope. As we only explore European countries, it may be the case that the health care systems are able to quickly upgrade their capacity to handle such a crisis. Alternatively, lower confidence societies may “give up” on the stay at home measures but continue to comply with handwashing and distancing requirements. Social/outgoing countries that also have low health care confidence may have strong pre-pandemic social norms and thus may be more susceptible to reversion. However, we also need to consider that the structure of each economy is different, with some unable to implement government support programs such as cash transfers and dole out programs, which are often more efficient and readily available in richer countries. Furthermore, it may be that people in countries with lower levels of confidence in the health care system (due to restricted availability of medical care) are less likely to be employed in work that can be carried out from home and may also have fewer resources to wait out the lockdown.

The mix of compliance behavior and fear of being dependent on the health care system is evident in the heatmaps. Regions with high levels of trust in the government but low levels of confidence in the health care system exhibit a strong increase in staying at home. In addition, when looking at policy strategies, we find that regions with higher confidence in the health care system are more likely to further reduce mobility once the government mandates its citizens not to leave home except for essential trips, compared to those with lower health care system confidence.

The main limitation of this study stems from the unclear nature of the question used to capture the dependent variable, which is framed by asking participants about their confidence in health care organizations. As mentioned in Sections 2.2 and 2.4, the health care system consists of at least two distinctly different parts, namely the people and the structure (organization), and it is not strictly clear to which of these parts the responses are related. The authors argue that in the vast majority of systems the health care workers are exceptional, and regardless of the resources or situation work above and beyond the normal call of duty to provide the highest possible quality care and support for every patient that comes before them. However, the same cannot be said of all health care systems (organizations), which are often under-resourced and underfunded, leaving the system without the capacity to administer health care in times of crisis when a large number of patients suddenly enter the system. Many systems were deluged with massive amounts of funding and fast-tracked policy revisions to prepare for the anticipated tsunami of COVID-19 patients; implementing rapid ICU training, sourcing mass numbers of ventilators (converting or construction), and building emergency field hospitals. We assume, but cannot be certain, that given the context and the framing of the WVS question regarding trust in a variety of organizations—ranging from public & private to non-profit institutions, responses were in reference to the capability of the health care system and not the quality of patient care–which in the authors’ opinions are representative of true selfless dedication and altruism of the highest order.

It is not uncommon for trust in general to deteriorate in situations of high uncertainty and ambiguous behavior [46], and the current situation is abundant in uncertainty and ambiguity. Unfortunately, the nature of the pandemic means that the stakes for cooperation and personal sacrifice are very high. Usually, the costs are limited in cases of individual non-compliance behavior (or defection) from the socially optimal course of action; for example, littering in a forest park only damages the immediate environment. However, due to the exponential risk of transmission, small instances of defection during a pandemic can have ramifications well beyond the individual, their community, or even their country. As Van Bavel et al. [24, p465] point out, “someone else’s infection is a threat to oneself and everyone else”. Thus, determining the factors that can improve compliance and cooperation remains important, especially in the initial months of a novel virus pandemic when treatment options are by definition experimental.

In general, these results highlight important pathways for how the architects of health policy can ensure societally optimal outcomes in relation to dealing with a pandemic. It is particularly useful in the case of societies with low confidence in their health care, where the additional strain of dealing with numerous infections would place the system under even greater pressure. Of course, compliance efficacy is determined by many factors; however, it is important to note the importance of health care confidence for heterogeneous societies when applying the recommendations of epidemiologists and public health experts. While human mobility is a useful tool in establishing a proxy for compliance to stay at home measures, we acknowledge that limitations exist with this form of measurement. In spite of these shortcomings, our findings indicate significant differences in behavior between high and low health care confidence societies, offering insights into the relative efficacy of existing policies and providing insights into potential future variations in communication and strategy in future health crises.

The relationship between trust in institutions, confidence in systems, and compliance decisions with respect to government authorities is a crucial element in human behavior. Fostering this connection is a reciprocal task of authorities and citizens–one requiring future engagement and investment. Improving this confidence through genuine upgrades and commitment to the quality of health care systems will offer long-term benefits beyond the in-the-moment health care received under normal circumstances; it will contribute to a foundation of trust and reciprocal cooperation, which is an investment that pays dividends during future health crises.

## Supporting information

S1 Table Countries and regions sample. (DOCX)

S2 Table Regression results on the control variables. (DOCX)

S3 Table Coefficients of institutional and governance quality and institutional and generalized trust on mobility change (control variables for models in S5 Fig). (DOCX)

S4 Table Regression results for mediation effect of trust in government. (DOCX)

S1 Fig Decrease in duration of non-residential mobility (daily) by regions with high and low levels of confidence in the health care system (a) over time (from 15 Feb to 5 June), (b) since the first confirmed case in the country, and (c) since the first confirmed death. For each region, we take the average of the percentage change in total number of visitors to locations classified as Retail & Recreation, Grocery & Pharmacy, Parks, Transit Stations, and Workplaces.
(DOCX)

S2 Fig Mobility change since first confirmed case in the country by regions with levels of confidence in health care system higher or lower than the corresponding country average. (DOCX)

S3 Fig Mobility pattern by regions with high and low levels of confidence in health care system. Regions are unweighted in contrast to Fig 1.
(DOCX)

S4 Fig Mobility patterns at the country level. (DOCX)

S5 Fig Estimated effect of confidence in health care system on mobility over time since first confirmed case, with various controls for institutional and governance quality and generalized and institutional trust. *Bureaucracy Quality*, *Democratic Accountability*, *Government Stability*, and *Law & Order* were obtained from the ICRG. *Trust (GPS)* was derived from the Global Preference Survey (Falk et al., 2016; Falk et al., 2018) by aggregating the measure into a region average. *Trust (WVS a* and *b)* were derived from the questions *Most people can be trusted* and *Trust*: *Other people in country* from the last available wave of the World Value Survey. Trust in *Government*, *Parliament*, *Political Parties*, *Civil Services*, and *Justice System/Courts* were also from the WVS, aggregated to the region average. All additional variables are standardized at the region level and recoded such that higher values indicate better governance quality or more trust.
(DOCX)

S6 Fig Effect of corruption and confidence in health care system on mobility. Blue indicates the estimated reduction in mobility change (difference in percentage increase in duration of staying home) in the second week since the first confirmed case compared to mobility change *one week before* the first case. Effects are predicted from the model at the 1^st^, 25^th^, 50^th^, 75^th^, and 99^th^ percentiles of the distribution of the two variables corruption index (ICRG) and confidence in health care system (EVS), which we categorized into five levels: *very low*, *low*, *neutral*, *high*, and v*ery high*, respectively. Statistical significance of the effect is indicated by the size of the markers.
(DOCX)

S7 Fig (DOCX)

S8 Fig Replication of Fig 2 with sample includes only countries for which there is an available 2017 EVS survey (5^th^ wave) measuring confidence in the healthcare system (*n =* 27). (DOCX)

S9 Fig Replication of Fig 3 with sample includes only countries for which there is an available 2017 EVS survey (5^th^ wave) measuring confidence in the healthcare system (*n =* 27). (DOCX)

S10 Fig Replication of Fig 4 with sample includes only countries for which there is an available 2017 EVS survey (5^th^ wave) measuring confidence in the healthcare system (*n =* 27). (DOCX)
