# Supplementary material for: How confidence in health care systems affects mobility and compliance during the COVID-19 pandemic
Source: PLoS One. 2020 Oct 15;15(10):e0240644. doi: 10.1371/journal.pone.0240644 (PMC7561184; doi:10.1371/journal.pone.0240644)
Supplement: S1 Table — (DOCX) [file pone.0240644.s001.docx]

**S1 Table.** Countries and Regions sample

| ISO2 | Country | Number of regions | Administrative level (Google-GADM) | European Values Study (EVS) wave |
| --- | --- | --- | --- | --- |
| AT | Austria | 9 | 1 | 2017 |
| BA | Bosnia and Herzegovina | 0 | 0 | 2017 |
| BE | Belgium | 3 | 1 | 2008 |
| BG | Bulgaria | 27 | 1 | 2017 |
| BY | Belarus | 0 | 0 | 2017 |
| CH | Switzerland^1^ | 20 | 1 | 2017 |
| CZ | Czech Republic | 14 | 1 | 2017 |
| DE | Germany | 16 | 1 | 2008 |
| DK | Denmark | 5 | 1 | 2017 |
| EE | Estonia | 5 | 1 | 2017 |
| ES | Spain | 19 | 1 | 2017 |
| FI | Finland^1^ | 18 | 2 | 2017 |
| FR | France | 13 | 1 | 2017 |
| GB | United Kingdom^1^ | 147 | 2 | 2017 |
| GE | Georgia | 0 | 0 | 2017 |
| GR | Greece | 7 | 1 | 2008 |
| HR | Croatia | 20 | 1 | 2017 |
| HU | Hungary | 20 | 1 | 2017 |
| IE | Ireland^1^ | 24 | 1 | 2008 |
| IT | Italy | 20 | 1 | 2017 |
| LT | Lithuania^1^ | 10 | 1 | 2017 |
| LU | Luxembourg | 0 | 0 | 2008 |
| LV | Latvia^2^ | 4 | 2 | 2008 |
| MD | Moldova | 0 | 0 | 2008 |
| MK | Macedonia | 0 | 0 | 2017 |
| MT | Malta | 0 | 0 | 2008 |
| NL | Netherlands | 12 | 1 | 2017 |
| NO | Norway^3^ | 11 | 1 | 2017 |
| PL | Poland | 16 | 1 | 2017 |
| PT | Portugal^1^ | 20 | 1 | 2008 |
| RO | Romania | 42 | 1 | 2017 |
| RS | Yugoslavia | 0 | 0 | 2017 |
| RU | Russia | 0 | 0 | 2017 |
| SE | Sweden^1^ | 21 | 1 | 2017 |
| SI | Slovenia^2^ | 15 | 2 | 2017 |
| SK | Slovak Republic | 8 | 1 | 2017 |
| TR | Turkey^1^ | 75 | 1 | 1999 |
| UA | Ukraine | 0 | 0 | 2008 |

^1^ Indicates that data from European Value Survey are on a broader regional level (e.g., for the UK, Google mobility data are available on administrative level similar to NUTS-3 division). ^2^ Due to insufficient volume of data to generate an aggregated and anonymous view of trends, mobility records for most municipalities of Latvia and regions of Slovenia are not available. ^3^ Pre-2020 region classification is used for Norway (e.g., Agder instead of Vest-Agder and Aust-Agder).
