# Supplementary material for: How confidence in health care systems affects mobility and compliance during the COVID-19 pandemic
Source: PLoS One. 2020 Oct 15;15(10):e0240644. doi: 10.1371/journal.pone.0240644 (PMC7561184; doi:10.1371/journal.pone.0240644)
Supplement: S2 Table — (DOCX) [file pone.0240644.s002.docx]

**S2 Table.** Regression results on the control variables

| Dependent variable | Change in duration of staying at home (%) | | | | | |
| --- | --- | --- | --- | --- | --- | --- |
|  | Confidence*Week since first case | | | Confidence*Week since first death | | |
| Independent variable | (C) | (CT) | (CTC) | (C) | (CT) | (CTC) |
| Weekends | -3.39^***^ | -3.52^***^ | -3.73^***^ | -3.54^***^ | -3.58^***^ | -3.54^***^ |
|  | (0.147) | (0.148) | (0.136) | (0.142) | (0.146) | (0.146) |
| Pandemic declared | 3.71^***^ | 1.04 | 9.94^***^ | 5.00^***^ | 2.05 | 9.16^***^ |
|  | (0.445) | (1.104) | (0.901) | (0.537) | (1.281) | (1.072) |
| *ln*(# confirmed cases+1) | 1.68^***^ | 1.35^***^ | 1.43^***^ | 1.21^***^ | 0.93^***^ | 0.99^***^ |
|  | (0.0686) | (0.0796) | (0.0791) | (0.0737) | (0.0823) | (0.0816) |
| Days after first death | -0.096^***^ | -0.043 | -0.32^***^ | -0.22^***^ | -0.13^***^ | -0.24^***^ |
|  | (0.0207) | (0.0285) | (0.0287) | (0.0258) | (0.0379) | (0.0377) |
| *ln*(GDP per capita) | 0.49 | 0.86 | -6.16^***^ | 2.08^***^ | 1.77^***^ | -2.60^***^ |
|  | (0.576) | (0.558) | (0.717) | (0.443) | (0.409) | (0.472) |
| Unemployment rate | 0.16^*^ | 0.12^†^ | -0.49^***^ | 0.13^*^ | 0.14^*^ | -0.44^***^ |
|  | (0.0744) | (0.0620) | (0.0827) | (0.0651) | (0.0581) | (0.0776) |
| Corruption | 0.84^*^ | 0.81^*^ | 8.67^***^ | 0.084 | 0.38 | 5.11^***^ |
|  | (0.388) | (0.369) | (0.645) | (0.364) | (0.324) | (0.358) |
| Population density | -0.0029^†^ | -0.0012 | -0.0067^***^ | 0.0025 | 0.0029^†^ | -0.0083^***^ |
|  | (0.00170) | (0.00166) | (0.00180) | (0.00151) | (0.00150) | (0.00177) |
| Urban population (%) | 0.039^*^ | 0.013 | -0.11^***^ | 0.028 | -0.0088 | -0.056^***^ |
|  | (0.0171) | (0.0164) | (0.0138) | (0.0185) | (0.0162) | (0.0120) |
| Population ages 65+ (%) | 0.037 | 0.16 | 0.70^***^ | -0.012 | 0.099 | 0.39^***^ |
|  | (0.139) | (0.124) | (0.0989) | (0.146) | (0.117) | (0.0903) |
| Female (% of total population) | 0.56^*^ | 0.52^*^ | -0.81^***^ | 0.39 | 0.54^*^ | -0.42^**^ |
|  | (0.235) | (0.210) | (0.137) | (0.277) | (0.219) | (0.132) |
| Average household size | -0.91 | 0.76 | 13.8^***^ | -0.66 | 0.31 | 6.50^***^ |
|  | (1.302) | (1.103) | (1.295) | (1.348) | (1.061) | (0.968) |
| Education index | -1.90 | -4.03 | 37.2^***^ | -7.78 | -5.99 | 19.7^***^ |
|  | (4.686) | (4.107) | (4.099) | (5.209) | (4.096) | (3.581) |
| Hospital beds (per 1,000) | -0.37^**^ | -0.36^***^ | -1.02^***^ | -0.30^**^ | -0.38^***^ | -1.30^***^ |
|  | (0.117) | (0.107) | (0.151) | (0.103) | (0.0894) | (0.136) |
| Out-of-pocket expenditure (%) | 0.027 | 0.055^*^ | 0.16^***^ | 0.073^**^ | 0.070^***^ | 0.15^***^ |
|  | (0.0320) | (0.0274) | (0.0392) | (0.0250) | (0.0207) | (0.0361) |
| Maximum temperature | -0.015^***^ | -0.016^***^ | -0.017^***^ | -0.017^***^ | -0.016^***^ | -0.017^***^ |
|  | (0.00101) | (0.00101) | (0.00100) | (0.00101) | (0.000962) | (0.000962) |
| Minimum temperature | -0.00030 | 0.00089 | 0.000087 | 0.0015 | 0.0024^**^ | 0.0016^†^ |
|  | (0.00115) | (0.00102) | (0.00102) | (0.00106) | (0.000931) | (0.000911) |
| Containment policies | Yes | Yes | Yes | Yes | Yes | Yes |
| Time FE | No | Yes | Yes | No | Yes | Yes |
| Country FE | No | No | Yes | No | No | Yes |
| Observations | 35690 | 35690 | 35690 | 35690 | 35690 | 35690 |
| Number of clusters | 597 | 597 | 597 | 597 | 597 | 597 |
| *R^2^*-between | 0.846 | 0.859 | 0.860 | 0.840 | 0.855 | 0.856 |
| *R^2^*-within | 0.771 | 0.800 | 0.889 | 0.753 | 0.800 | 0.892 |
| *R^2^*-overall | 0.822 | 0.839 | 0.857 | 0.811 | 0.836 | 0.854 |

Notes: Standard errors clustered on the regional level in parentheses. † *p* < .10; * *p* < .05; ** *p* < .01; *** *p* < .001.
