# Supplementary material for: How confidence in health care systems affects mobility and compliance during the COVID-19 pandemic
Source: PLoS One. 2020 Oct 15;15(10):e0240644. doi: 10.1371/journal.pone.0240644 (PMC7561184; doi:10.1371/journal.pone.0240644)
Supplement: S3 Table — (DOCX) [file pone.0240644.s003.docx]

**S3 Table.** Coefficients of institutional and governance quality and institutional and generalized trust on mobility change (control variables for models in S5 Fig).

|  | Control variables | *b* | *s.e.* | N | Cluster |
| --- | --- | --- | --- | --- | --- |
| (1) | Bureaucracy Quality | 2.55*** | (0.304) | 35690 | 597 |
| (2) | Democratic Accountability | 0.57 | (0.571) | 35690 | 597 |
| (3) | Government Stability | 0.43* | (0.195) | 35690 | 597 |
| (4) | Law & Order | 0.29 | (0.404) | 35690 | 597 |
| (5) | Trust (GPS) | -0.10 | (0.170) | 27237 | 433 |
| (6) | Trust (WVS a) | -0.39* | (0.153) | 35767 | 598 |
| (7) | Trust (WVS b) | 0.45* | (0.227) | 27111 | 472 |
| (8) | Government | -0.39* | (0.174) | 35767 | 598 |
| (9) | Parliament | 0.30† | (0.173) | 35767 | 598 |
| (10) | Political Parties | -0.63** | (0.241) | 35767 | 598 |
| (11) | Civil Services | 0.19 | (0.194) | 35767 | 598 |
| (12) | Justice System/Courts | -0.38† | (0.206) | 35767 | 598 |

*Notes*: Dependent variable: Duration change in staying home. In each specification, we include the interaction terms between confidence in health care system and weeks since first confirmed case (estimates presented in S5 Fig) and control for all variables listed in model (C) from S2 Table except for *Corruption*. All variables listed are standardized at the region level and recoded such that higher values indicate better governance quality or more trust. Standard errors clustered at regional level in parentheses. † *p* < .10; * *p* < .05; ** *p* < .01; *** *p* < .001.
