# Supplementary material for: How confidence in health care systems affects mobility and compliance during the COVID-19 pandemic
Source: PLoS One. 2020 Oct 15;15(10):e0240644. doi: 10.1371/journal.pone.0240644 (PMC7561184; doi:10.1371/journal.pone.0240644)
Supplement: S4 Table — (DOCX) [file pone.0240644.s004.docx]

**S4 Table.** Regression results for mediation effect of trust in government.

|  | β | s.e. |
| --- | --- | --- |
| Confidence in healthcare system | 1.60^†^ | (0.887) |
| Trust in government | 0.93 | (1.233) |
| First week after 1^st^ confirmed case | -1.22 | (2.335) |
| Second week after 1^st^ confirmed case | -6.75 | (4.176) |
| Healthcare * Trust in gov | -0.78^†^ | (0.435) |
| First week*Healthcare | -0.69 | (0.829) |
| Second week*Healthcare | 1.35 | (1.566) |
| First week * Trust in gov | 0.56 | (1.258) |
| Second week* Trust in gov | 6.33^**^ | (2.197) |
| First week * Healthcare * Trust in gov | 0.21 | (0.435) |
| Second week* Healthcare * Trust in gov | -1.79^*^ | (0.800) |
| Weekends | -0.67^***^ | (0.0789) |
| Pandemic declared | 1.80^***^ | (0.221) |
| *ln*(# confirmed cases+1) | 0.74^***^ | (0.0777) |
| Days after first death | -0.088^***^ | (0.0199) |
| lnwdi_gdpcapcon2010 | -0.70 | (0.515) |
| Unemployment, total (% of total labor force) (modeled ILO) | -0.085^†^ | (0.0488) |
| Corruption (F) | 1.77^***^ | (0.411) |
| Population density (people per sq. km of land area) | 0.0015^†^ | (0.000804) |
| Urban population (% of total population) | 0.0052 | (0.0105) |
| Population ages 65 and above (% of total population) | 0.39^***^ | (0.0951) |
| Population, female (% of total population) | -0.45^***^ | (0.114) |
| Average household size (number of members) | 5.47^***^ | (0.796) |
| Education index | 15.1^***^ | (2.488) |
| Hospital beds (per 1,000 people) | -0.043 | (0.0823) |
| Out-of-pocket expenditure (% of current health expenditure) | 0.15^***^ | (0.0173) |
| Maximum temperature (tenths of degrees C); mean | -0.0098^***^ | (0.00121) |
| Minimum temperature (tenths of degrees C); mean | 0.0057^***^ | (0.000959) |
| Containment policies | Yes |  |
| Observations | 5740 |  |
| Number of clusters | 377 |  |
| *R^2^*-between | 0.822 |  |
| *R^2^*-within | 0.878 |  |
| *R^2^*-overall | 0.836 |  |

*Notes*: Dependent variable: Duration change in staying home. Standard errors clustered at regional level in parentheses. † *p* < .10; * *p* < .05; ** *p* < .01; *** *p* < .001.
