# Supplementary material for: How confidence in health care systems affects mobility and compliance during the COVID-19 pandemic
Source: PLoS One. 2020 Oct 15;15(10):e0240644. doi: 10.1371/journal.pone.0240644 (PMC7561184; doi:10.1371/journal.pone.0240644)

**S1 Fig. Decrease in duration of non-residential mobility (daily) by regions with high and low levels of confidence in the health care system (a) over time (from 15 Feb to 5 June), (b) since the first confirmed case in the country, and (c) since the first confirmed death**. For each region, we take the average of the percentage change in total number of visitors to locations classified as *Retail & Recreation*, *Grocery & Pharmacy*, *Parks*, *Transit Stations*, and *Workplaces*.


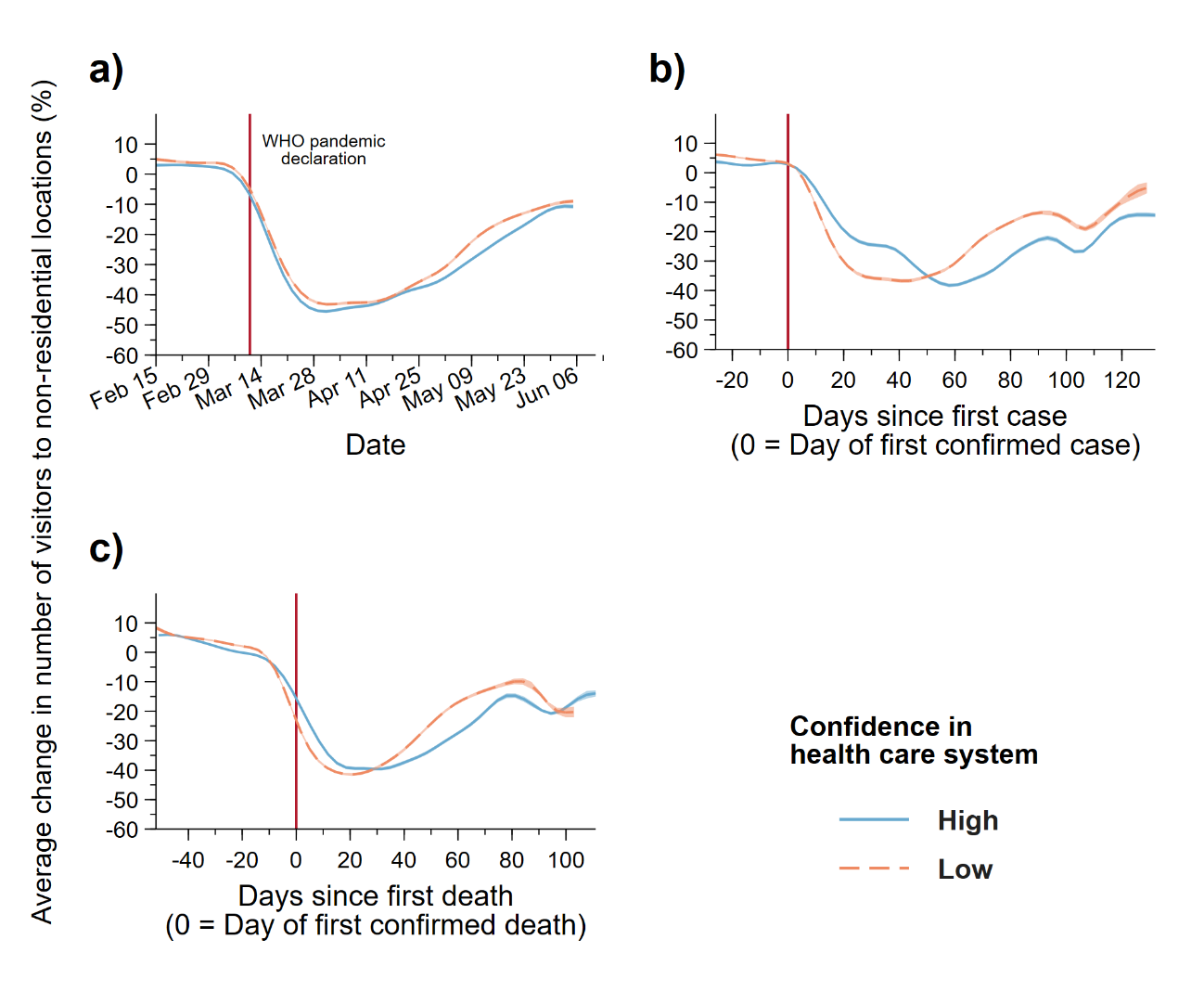

Supplement: S1 Fig — Decrease in duration of non-residential mobility (daily) by regions with high and low levels of confidence in the health care system (a) over time (from 15 Feb to 5 June), (b) since the first confirmed case in the country, and (c) since the first confirmed death. For each region, we take the average of the percentage change in total number of visitors to locations classified as Retail & Recreation, Grocery & Pharmacy, Parks, Transit Stations, and Workplaces. (DOCX) [file pone.0240644.s005.docx]
