# Supplementary material for: How confidence in health care systems affects mobility and compliance during the COVID-19 pandemic
Source: PLoS One. 2020 Oct 15;15(10):e0240644. doi: 10.1371/journal.pone.0240644 (PMC7561184; doi:10.1371/journal.pone.0240644)

**S2 Fig. Mobility change since first confirmed case in the country by regions with levels of confidence in health care system higher or lower than the corresponding country average**.


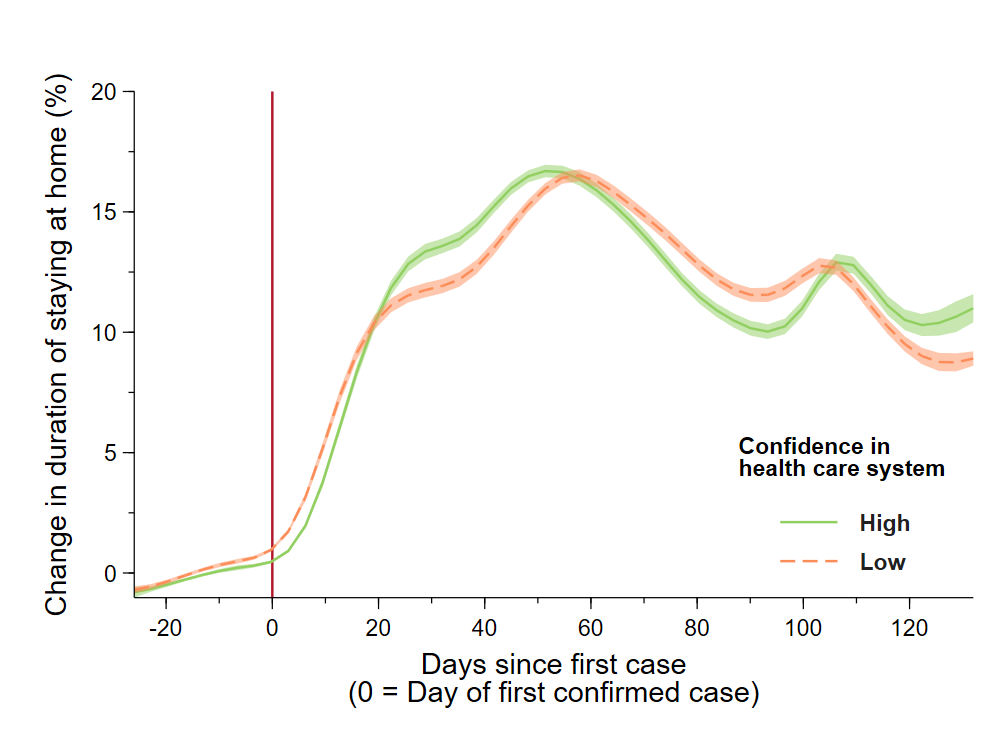

Supplement: S2 Fig — (DOCX) [file pone.0240644.s006.docx]
