# Supplementary material for: How confidence in health care systems affects mobility and compliance during the COVID-19 pandemic
Source: PLoS One. 2020 Oct 15;15(10):e0240644. doi: 10.1371/journal.pone.0240644 (PMC7561184; doi:10.1371/journal.pone.0240644)
Supplement: S3 Fig — Regions are unweighted in contrast to Fig 1. (DOCX) [file pone.0240644.s007.docx]

**S3 Fig.** **Mobility pattern by regions with high and low levels of confidence in health care system**. Regions are unweighted in contrast to Fig 1.

**
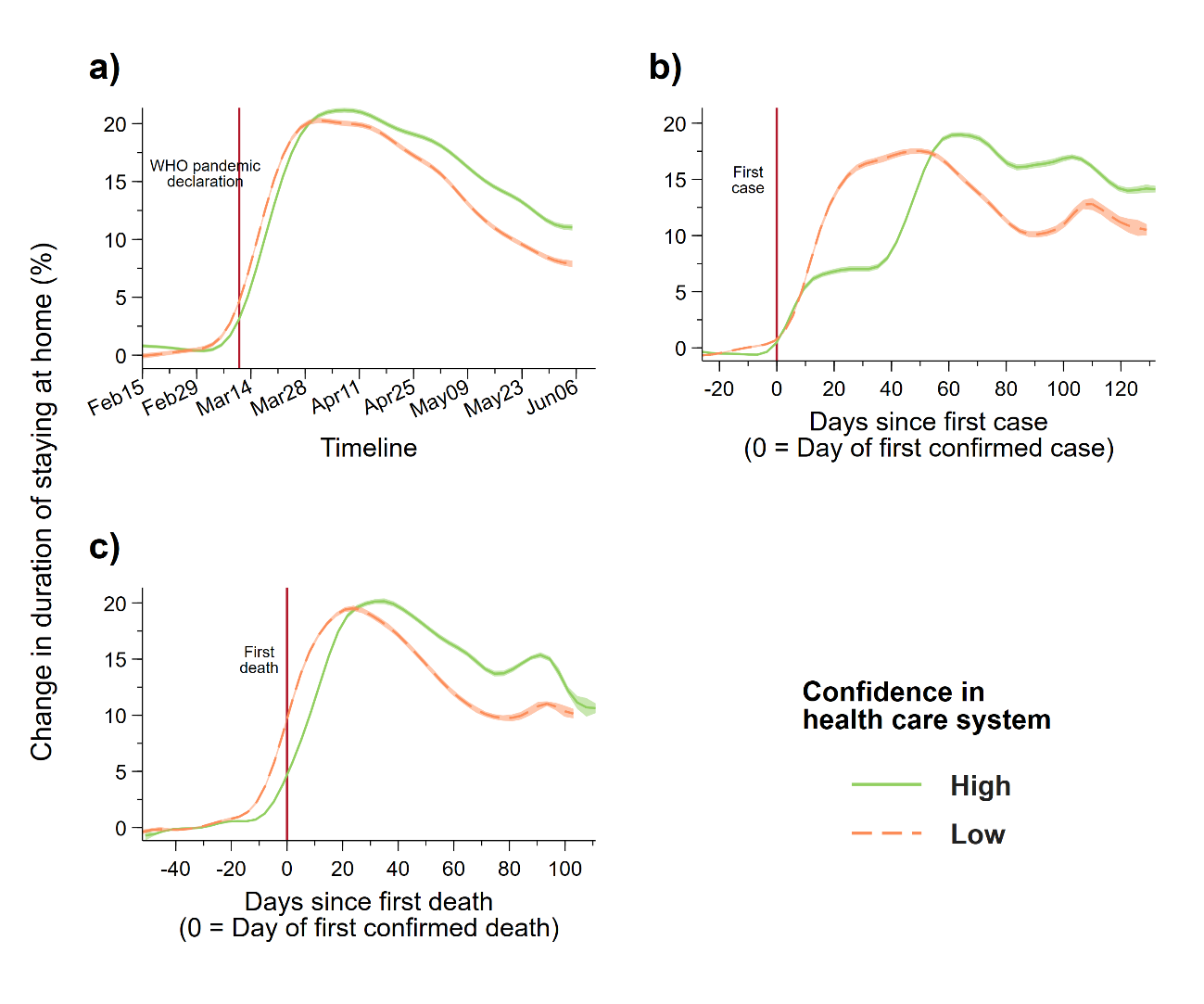
**
