# Supplementary figures and images for: How confidence in health care systems affects mobility and compliance during the COVID-19 pandemic
Source: PLoS One. 2020 Oct 15;15(10):e0240644. doi: 10.1371/journal.pone.0240644 (PMC7561184; doi:10.1371/journal.pone.0240644)

**S4 Fig. Mobility patterns at the country level**.


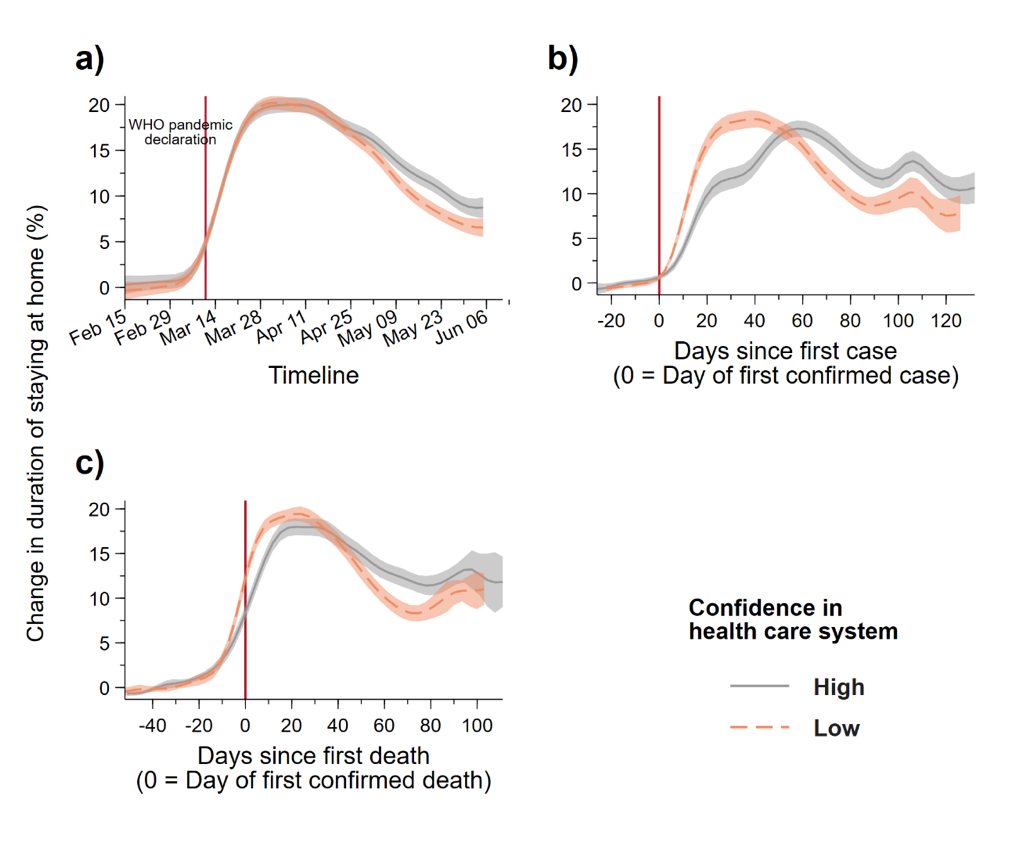

Supplement: S4 Fig — (DOCX) [file pone.0240644.s008.docx]

**S7 Fig.**


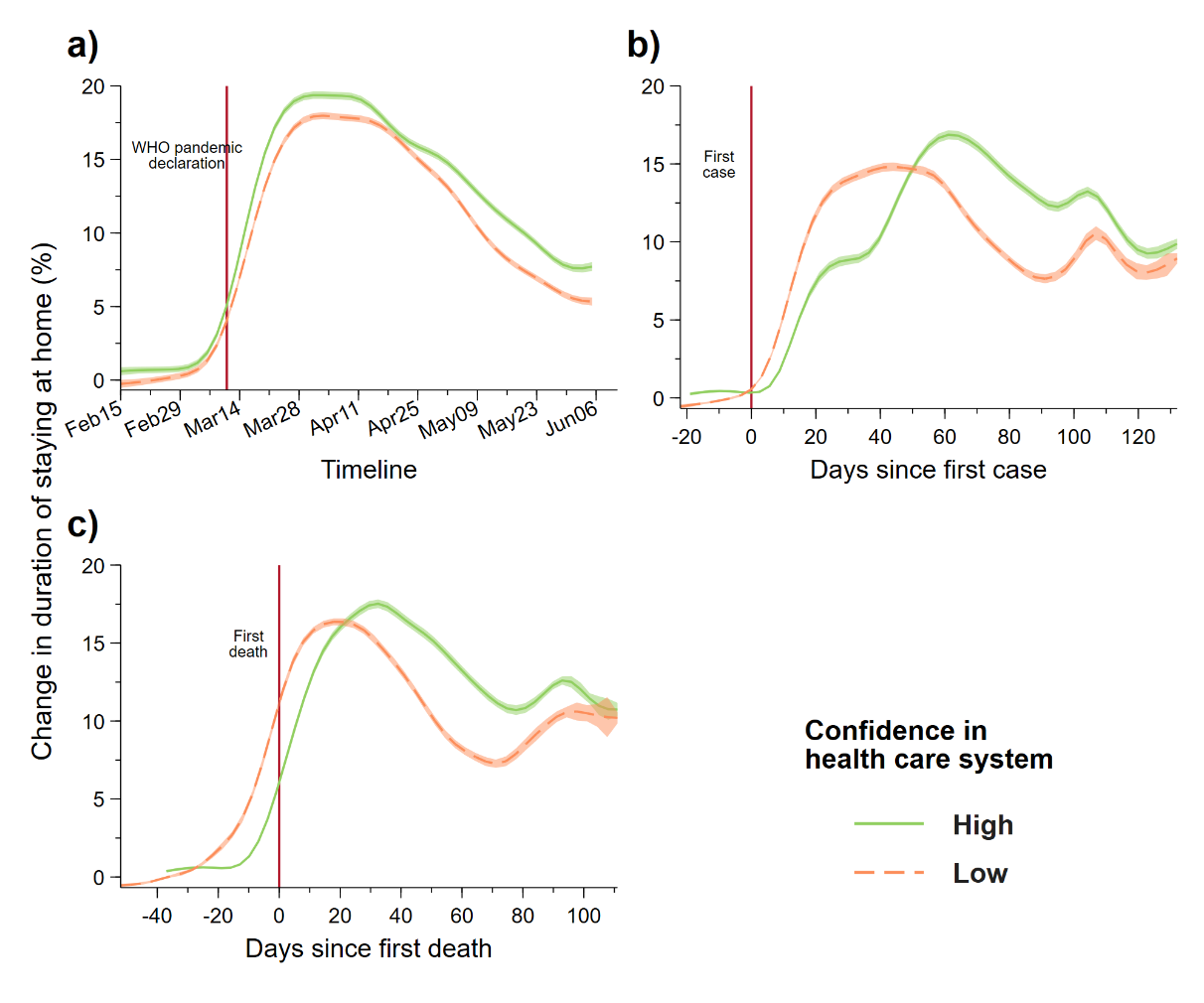

Supplement: S7 Fig — (DOCX) [file pone.0240644.s011.docx]
