# Supplementary material for: How confidence in health care systems affects mobility and compliance during the COVID-19 pandemic
Source: PLoS One. 2020 Oct 15;15(10):e0240644. doi: 10.1371/journal.pone.0240644 (PMC7561184; doi:10.1371/journal.pone.0240644)
Supplement: S5 Fig — Bureaucracy Quality, Democratic Accountability, Government Stability, and Law & Order were obtained from the ICRG. Trust (GPS) was derived from the Global Preference Survey (Falk et al., 2016; Falk et al., 2018) by aggregating the measure into a region average. Trust (WVS a and b) were derived from the questions Most people can be trusted and Trust: Other people in country from the last available wave of the World Value Survey. Trust in Government, Parliament, Political Parties, Civil Services, and Justice System/Courts were also from the WVS, aggregated to the region average. All additional variables are standardized at the region level and recoded such that higher values indicate better governance quality or more trust. (DOCX) [file pone.0240644.s009.docx]

**S5 Fig. Estimated effect of confidence in health care system on mobility over time since first confirmed case, with various controls for institutional and governance quality and generalized and institutional trust**. *Bureaucracy Quality, Democratic Accountability, Government Stability,* and *Law & Order* were obtained from the ICRG. *Trust (GPS)* was derived from the Global Preference Survey (Falk et al., 2016; Falk et al., 2018) by aggregating the measure into a region average. *Trust* *(WVS a* and *b)* were derived from the questions *Most people can be trusted* and *Trust: Other people in country* from the last available wave of the World Value Survey. Trust in *Government, Parliament, Political Parties, Civil Services,* and *Justice System/Courts* were also from the WVS, aggregated to the region average. All additional variables are standardized at the region level and recoded such that higher values indicate better governance quality or more trust.

**
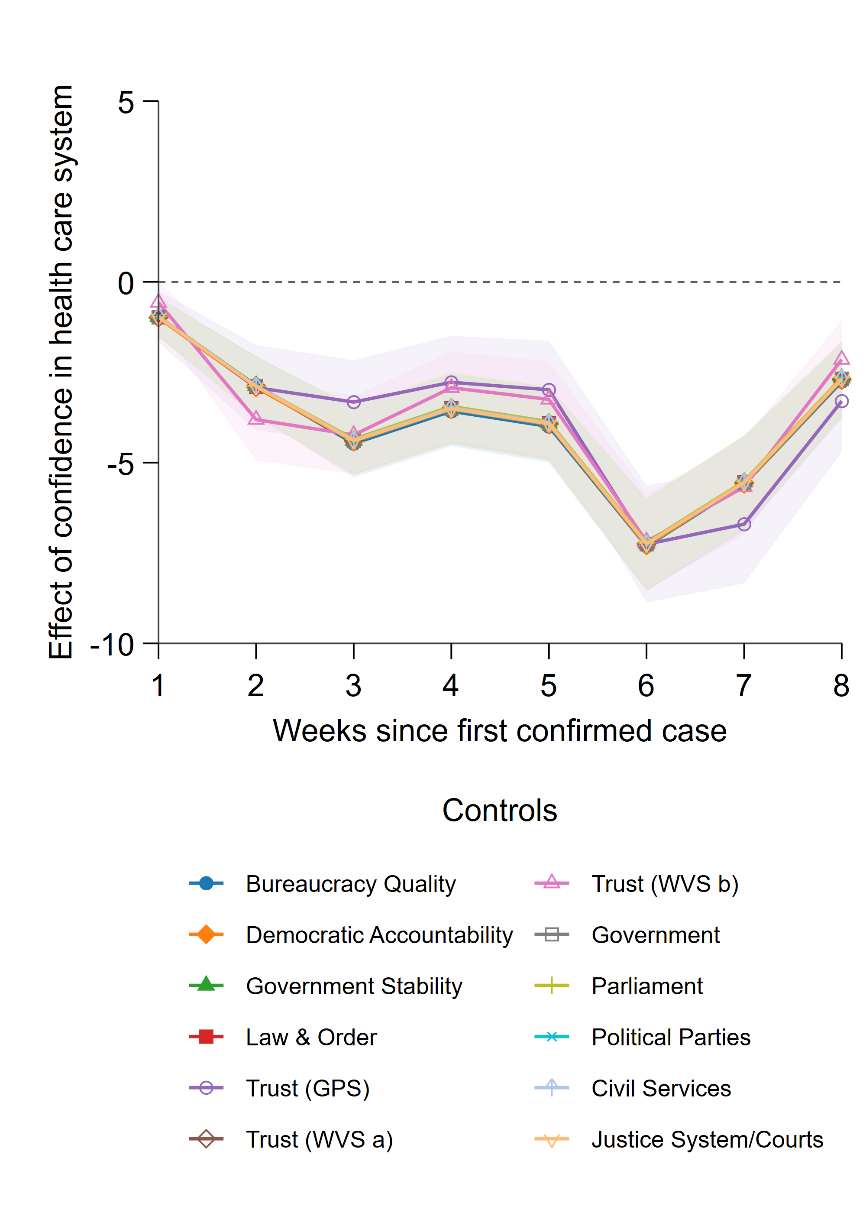
**
