# Supplementary material for: How confidence in health care systems affects mobility and compliance during the COVID-19 pandemic
Source: PLoS One. 2020 Oct 15;15(10):e0240644. doi: 10.1371/journal.pone.0240644 (PMC7561184; doi:10.1371/journal.pone.0240644)

**S6 Fig. Effect of corruption and confidence in health care system on mobility.** Blue indicates the estimated reduction in mobility change (difference in percentage increase in duration of staying home) in the second week since the first confirmed case compared to mobility change *one week before* the first case*.* Effects are predicted from the model at the 1^st^, 25^th^, 50^th^, 75^th^, and 99^th^ percentiles of the distribution of the two variables corruption index (ICRG) and confidence in health care system (EVS), which we categorized into five levels: *very low*, *low*, *neutral*, *high*, and v*ery high*, respectively. Statistical significance of the effect is indicated by the size of the markers.


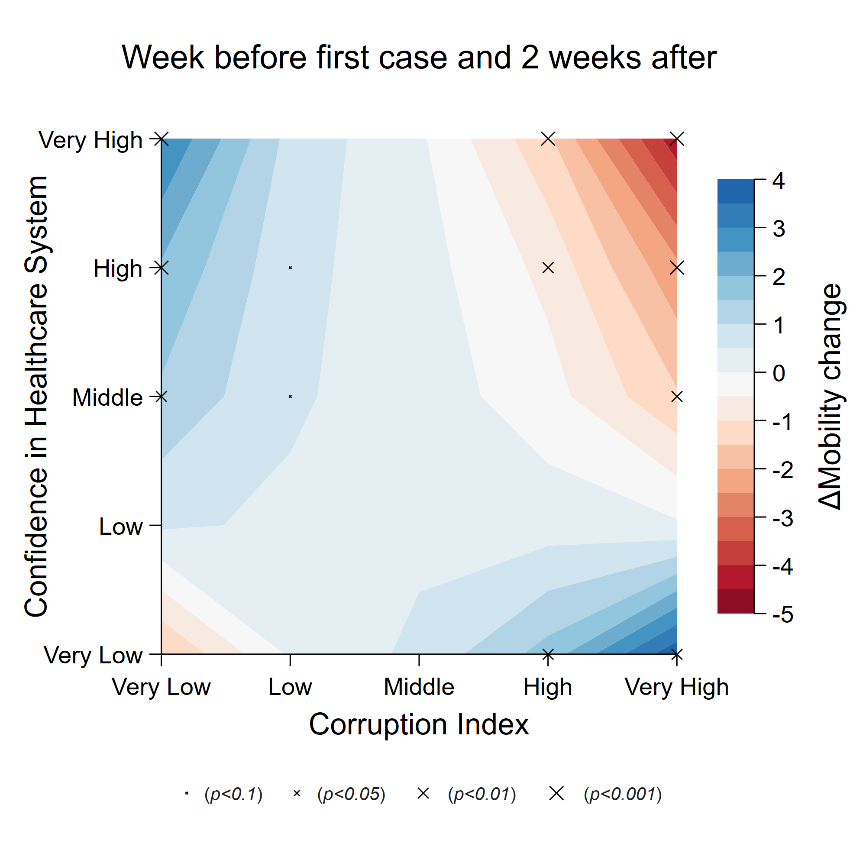

Supplement: S6 Fig — Blue indicates the estimated reduction in mobility change (difference in percentage increase in duration of staying home) in the second week since the first confirmed case compared to mobility change one week before the first case. Effects are predicted from the model at the 1st, 25th, 50th, 75th, and 99th percentiles of the distribution of the two variables corruption index (ICRG) and confidence in health care system (EVS), which we categorized into five levels: very low, low, neutral, high, and very high, respectively. Statistical significance of the effect is indicated by the size of the markers. (DOCX) [file pone.0240644.s010.docx]
