# Supplementary material for: How confidence in health care systems affects mobility and compliance during the COVID-19 pandemic
Source: PLoS One. 2020 Oct 15;15(10):e0240644. doi: 10.1371/journal.pone.0240644 (PMC7561184; doi:10.1371/journal.pone.0240644)
Supplement: S10 Fig — (DOCX) [file pone.0240644.s014.docx]

**S10 Fig. Replication of Fig 4 with sample includes only countries for which there is an available 2017 EVS survey (5^th^ wave) measuring confidence in the healthcare system (*n=*27).**

**
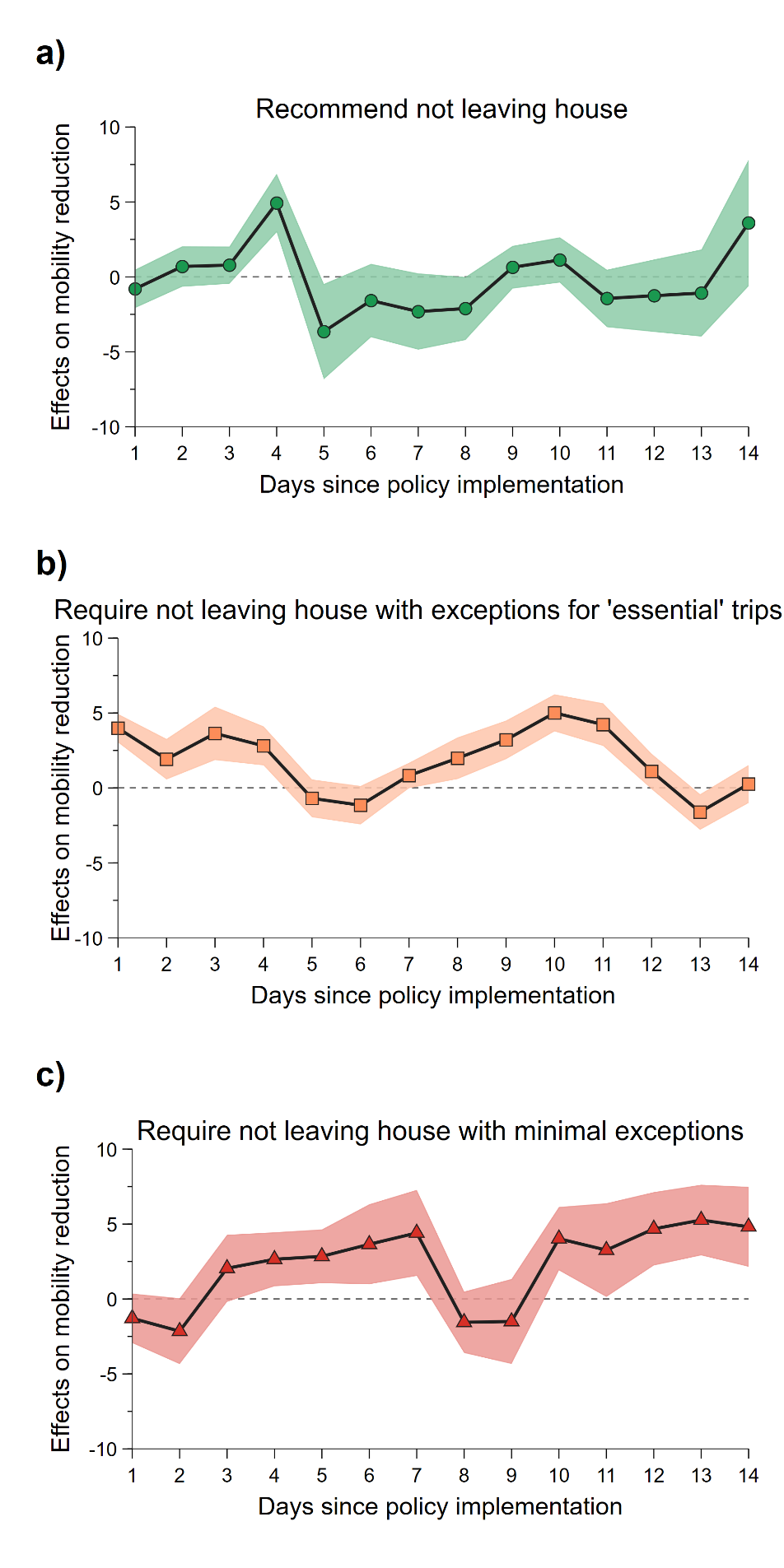
**
